# Size, age, and habitat determine effectiveness of Palau's Marine Protected Areas

**DOI:** 10.1371/journal.pone.0174787

**Published:** 2017-03-30

**Authors:** Alan M. Friedlander, Yimnang Golbuu, Enric Ballesteros, Jennifer E. Caselle, Marine Gouezo, Dawnette Olsudong, Enric Sala

**Affiliations:** 1 Pristine Seas, National Geographic Society, Washington, DC, United States of America; 2 Fisheries Ecology Research Laboratory, University of Hawaii, Honolulu, Hawaii, United States of America; 3 Palau International Coral Reef Center, Koror, Palau; 4 Centre d'Estudis Avançats de Blanes (CEAB-CSIC), Blanes, Spain; 5 Marine Science Institute, University of California Santa Barbara, Santa Barbara, California, United States of America; Department of Agriculture and Water Resources, AUSTRALIA

## Abstract

Palau has a rich heritage of conservation that has evolved from the traditional moratoria on fishing, or “bul”, to more western Marine Protected Areas (MPAs), while still retaining elements of customary management and tenure. In 2003, the Palau Protected Areas Network (PAN) was created to conserve Palau’s unique biodiversity and culture, and is the country’s mechanism for achieving the goals of the Micronesia Challenge (MC), an initiative to conserve ≥30% of near-shore marine resources within the region by 2020. The PAN comprises a network of numerous MPAs within Palau that vary in age, size, level of management, and habitat, which provide an excellent opportunity to test hypotheses concerning MPA design and function using multiple discreet sampling units. Our sampling design provided a robust space for time comparison to evaluate the relative influence of potential drivers of MPA efficacy. Our results showed that no-take MPAs had, on average, nearly twice the biomass of resource fishes (i.e. those important commercially, culturally, or for subsistence) compared to nearby unprotected areas. Biomass of non-resource fishes showed no differences between no-take areas and areas open to fishing. The most striking difference between no-take MPAs and unprotected areas was the more than 5-fold greater biomass of piscivorous fishes in the MPAs compared to fished areas. The most important determinates of no-take MPA success in conserving resource fish biomass were MPA size and years of protection. Habitat and distance from shore had little effect on resource fish biomass. The extensive network of MPAs in Palau likely provides important conservation and tourism benefits to the Republic, and may also provide fisheries benefits by protecting spawning aggregation sites, and potentially through adult spillover.

## Introduction

Palau has a rich tradition of fisheries management and stewardship of its waters [1–4]. Traditionally, Palau had strong community control that closed areas to fishing through implementation of traditional moratoria on fishing, or “bul”, prohibiting all use for a restricted period, but usually not indefinitely [5–7]. This localized adaptive management was based on customary knowledge and practices, and was responsive to changes in resource abundance [1].

Conservation in Palau has evolved from the traditional “bul” to more western Marine Protected Areas (MPAs). The government of Palau was instrumental in establishing the Micronesia Challenge–a conservation initiative to protect >30% of the marine ecosystems of the region by 2020 through the establishment of local Protected Areas Network (PAN) [8–10]. The PAN was established by national law in 2003, and created a framework for a national system of marine and terrestrial protected areas. Currently, there are 35 MPAs throughout Palau, encompassing all major habitat types, ranging from nearshore mangroves and seagrass beds to offshore coral reefs, with > 45% of the country's nearshore waters under some form of protection [11–13]. These MPAs range in management from complete no-take to subsistence fishing only, and not all are included in the PAN.

The people of Palau and other tropical island nations rely heavily on coral reefs for the ecosystem services they provide, such as protection from storms, food provisioning, perpetuation of cultural practices, and revenue from tourism [4, 14–16]. Palau is one of the world’s top dive destinations, with tourists coming to experience its high biodiversity and unique marine ecosystems [17–19]. In recent years, tourism has contributed roughly three quarters of GDP growth, more than 80% of exports of goods and services, 15% of total tax revenue, and 40% of total employment [20].

Due to local and global threats, coral reefs are becoming increasingly degraded worldwide, necessitating better conservation and management measures [21–23]. MPAs have proven to be an effective ecosystem-based management tool to conserve biodiversity and manage fisheries [24–26]. By protecting populations, habitats, and ecosystems within their borders, no-take MPAs provide a spatial refuge for the entire ecological system they contain and provide a powerful buffer against anthropogenic effects and natural variability [27–30]. In addition to resource management, MPAs also contribute to the long-term livelihoods of island people though the strong cultural and economic connections between islanders and the sea, as well as their interdependence on a healthy marine environment for survival and prosperity [31].

The effectiveness of MPAs can be influenced by their size, shape, age, level of protection, and the movement patterns of individual species [32–36]. Fully protected areas have been shown to have much greater conservation benefits compared with areas under lesser levels of protection [37]. It is assumed that larger MPAs are more effective because they protect a greater amount and diversity of habitats, and encompass and protect critical habitats or processes that maintain populations and ecosystem stability, which provides protection for a wider range of species and buffers against losses associated with environmental fluctuations and large-scale disturbances [38–41]. Large MPAs are more likely to contain fully functional ecosystems and suffer less from outside effects since they have a smaller perimeter-to-area ratio [42–43].

While several meta-analyses of MPAs have not shown an effect of reserve size [44–45], these studies contained relatively few large no-take areas, and the wide range of locations and biogeographic affinities examined may mask the effects of MPA size [45]. A meta-analysis of 19 European no-take MPAs found that for every 1-fold increase in no-take MPA size, there was a 35% increase in the density of commercial fishes [35]. Edgar and Barrett [46] compared four no-take MPAs in Tasmania with unprotected reference regions and found that the largest MPA had higher fish species richness, higher density of large fish, and larger-sized exploitable fishes when compared with fished reference sites.

Decadal-scale observations of no-take MPAs have shown direct effects on target species typically occurring within 5 years, with most target species showing initial direct effects, but their trajectories were highly variable based on the life history characteristics of the species examined [36, 47–48]. The average time for indirect effects that occur through cascading trophic interactions took 13 years or more to develop [36], and many non-fishery species did not show any response to protection at all [48]. A study of MPAs in eastern Australia showed that many of the targeted taxa examined were more abundant in large no-take MPAs within a few years of the establishment compared with the small no-take MPAs and the fished sites [49]. Collectively, these studies show that MPA effects can be slow, complex, and species-specific.

The objectives of this study were to examine the effectiveness of Palau’s MPAs relative to comparable fished areas, and to determine which factors lead to better success among these MPAs. A subset of Palau’s MPAs have been monitored for a number of years, but prior to our study no comprehensive evaluation of the efficacy of these MPAs has been conducted. We used integrated survey methods, across multiple taxonomic groups, conducted at the same time to compare these MPAs to one another and to comparable adjacent habitats. This approach provided a robust comparison among these MPAs and between these MPAs and reference areas, and while it represents a snapshot in time, this work complements the information currently being collected over a longer time period.

## Methods

### Ethics statement

Data were collected by all authors in a collaborative effort. Non-invasive research was conducted, which included photographs and visual estimates described in the methods. The Republic of Palau granted all necessary permission to conduct this research. No vertebrate sampling was conducted and therefore no approval was required by the University of Hawaii Institutional Animal Care and Use Committee. Our data are available at Data Dryad: doi:10.5061/dryad.tp3j5.

**Fig 1:**
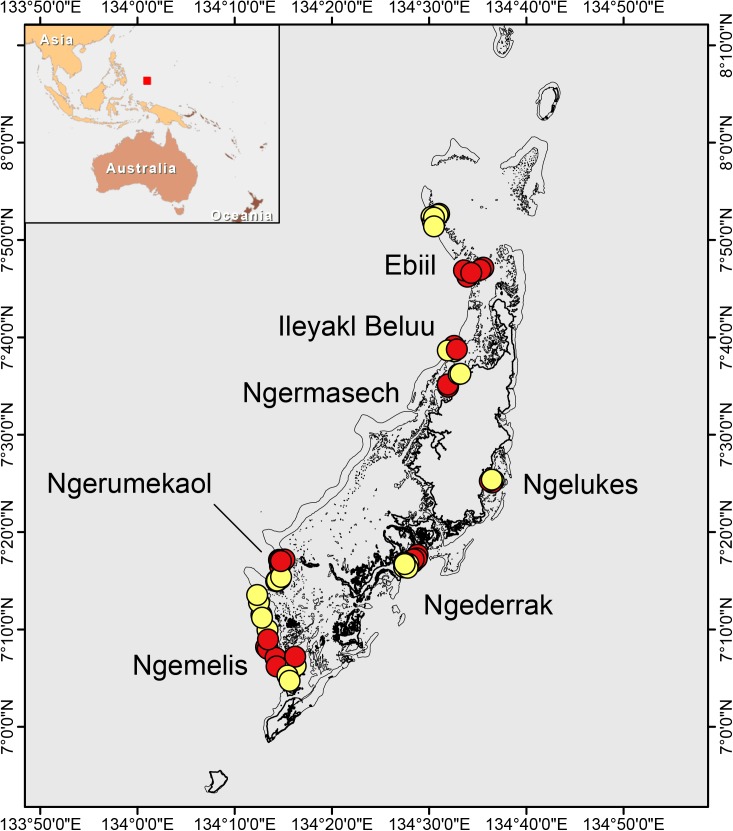
Locations of the Marine Protected Areas (red) and adjacent open sites (yellow) in Palau.

Of the 35 MPAs within the PAN, many protect nearshore mangrove, estuary, or seagrass habitats, while others are species-specific (e.g., clams, crabs) management areas, or remote atolls. We examined a subset of MPAs within the Palau PAN that were completely no-take areas, except for Ngemelis, which prohibits fishing within dive and snorkel sites and was considered as no-take for this study. We compared ecosystem characteristics within these areas to similar adjacent unprotected habitats (Fig 1). In the case of Ebiil, the control site was ~ 10 km to the north to incorporate comparable channel habitats. Previously created digital benthic habitat maps for all MPAs and adjacent habitats [50] were used to create a spatially-explicit stratified, random sampling design. Habitat features were mapped by visually interpreting multispectral satellite imagery and random sampling points were assigned within the major hard bottom geomorphic strata (e.g., forereef, patch reefs, channels) common to the MPA and their adjacent area. All adjacent area samples were > 500 m from the nearest MPA boundary. The MPAs ranged in age from 17 to 38 years of protection and from 0.4 km^2^ to 40 km^2^ in size (Table 1). The size range of these MPAs was representative of most of the MPAs within the PAN (range: 0.04–98.00 km^2^, median = 0.90 km^2^). All surveys were conducted in September 2014.

**Table 1:** Characteristics of Marine Protected Areas in Palau surveyed during the 2014 expedition. N is the number of transects at each MPA, divided equally between the two depth strata (10 and 20 m). An equal number of samples were conducted at adjacent areas open to fishing.

Name	State	Year est.	Size (km^2^)	N benthos	N fishes	Habitat tabitatypes	Restrictions
Ebiil	Ngerchelong	1999	37.9	12	36	Reef, channel	No fishing
Ngermasech	Ngardmau	1998	3.3	4	12	Mangrove, seagrass, coral reef	No entry, no fishing
Ngederrak	Koror	2001	5.9	12	36	Seagrass & reef flat	No entry, no fishing
Ngerumekaol	Koror	1976	3.5	12	36	Reef	No fishing
Ngemelis	Koror	1995	40.3	16	48	Islands & reefs	No fishing w/in dive & snorkel sites
Ngelukes	Ngchesar	2002	1.0	4	12	Patch reef	No entry, no fishing
Ileyakl Beluu	Ngardmau	2005	0.4	4	12	Reef	No entry, no fishing

### Benthos

Characterization of the benthos was conducted along 50 m-long transects oriented parallel to the shoreline at two depth strata (20 and 10 m). For algae, corals, and other sessile invertebrates, we used a line-point intercept methodology along each transect, recording the species or taxa found every 20 cm on the measuring tape. Benthic organisms were identified to the lowest possible taxonomic level, with overall benthic cover classified into major functional groupings (hard coral, soft coral, bare substrate, turf algae, macroalgae, blue-green algae, crustose coralline algae [CCA], soft sediment, seagrass, and sponge) for analyses.

### Fishes

At each of two depth strata within a site (20 and 10 m), divers counted and estimated lengths for select fishes (see below for details) encountered within fixed-length (25-m) belt transects whose widths differed depending on direction of swim. All fish ≥ 20 cm total length (TL) were tallied within a 4-m wide strip surveyed on an initial “swim-out” as the transect line was laid (transect area = 100 m^2^). All fishes < 20 cm TL were tallied within a 2-m wide strip surveyed on the return swim back along the laid transect line (transect area = 50 m^2^). The fish survey was limited to species from 17 families, which comprised most of the fish biomass on the reef and were important fisheries or ecological species (Acanthuridae, Caesionidae, Carangidae, Carcharhinidae, Haemulidae, Kyphosidae, Labridae, Lethrinidae, Lutjanidae, Mullidae, Muraenidae, Scaridae, Scombridae, Serranidae, Siganidae, Sphyraenidae, Zanclidae) (S1 Table). This dataset resulted in density and length estimates for 165 species and of these, 139 (from 15 families) were considered primary targeted resource species. These were species important for commercial, cultural, or subsistence fishing in Palau based on discussions with local fishers, scientists, and resource managers.

The survey methodology was designed to minimize bias associated with *in situ* underwater visual censuses [51]. Constraints on the focal window size and survey duration for the swim-out limited problems of over-counting large-bodied, vagile species. Use of 2 transect areas (4-m vs. 2-m lanes) compensated for some of the size-specific differences in density, namely that larger-bodied fish are typically less abundant than their smaller-bodied counterparts, addressing some concerns of differing patterns of variance across size classes [52].

The biomass of individual fishes was estimated using the allometric length-weight conversion: W = aTL^b^, where parameters a and b are species-specific constants, TL is total length in cm, and W is weight in grams. Length-weight fitting parameters were obtained from FishBase [53]. The sum of all individual weights and numerical densities was used to estimate biomass density by species. Fishes were categorized into four trophic groups (piscivore, herbivore, secondary consumer, and planktivore) based on published literature.

### Statistical analyses

Benthic community composition among MPAs and adjacent open areas was compared using permutation-based multivariate analysis of variance (PERMANOVA, PRIMER v6, [54]). A Bray–Curtis similarity matrix was created from percent cover of major benthic components and arcsine square root transformed prior to conducting the PERMANOVA. Management (MPA vs. open) was treated as a fixed factor and location was nested within management and treated as a random factor. Similarity of Percentages (SIMPER) was used to determine the benthic functional groups most responsible for the percentage dissimilarities between management regimes (MPA vs. open) using Bray-Curtis similarity analysis of hierarchical agglomerative group average clustering [55].

To explore the gradients in benthic community structure among sites, we performed a principal components analysis (PCA) on the percent cover of major benthic functional groups. Data were arcsine square root transformed to conform to the assumptions of the PCA. Non-metric multi-dimensional scaling (nMDS) analysis was conducted using PRIMER v6 [54] to examine differences in resource fish biomass among locations and between management regimes. A Bray–Curtis similarity matrix was constructed based on resource fish biomass, which was square root transformed prior to analysis.

Percent live coral cover was compared among locations and between management regimes using a generalized linear model (GLM) with a normal distribution and identity link function. Management (MPA vs. open) was treated as a fixed factor and locations were nested within management. Data were arcsine square root transformed prior to analysis. Percent live coral cover between MPA and open pairs of sites were tested using contrasts of the least squares means. Resource and non-resource fish biomass was compared using a GLM with a Poisson distribution and log link function, with contrasts between inside and outside MPAs performed as described above. Fish trophic biomass among locations was compared in a similar manner. All GLM analyses were performed using JMP Pro 12.2 [56].

To describe the pattern of fish trophic structure within MPAs and their relationship to MPA characteristics, we performed direct gradient analysis (redundancy analysis: RDA) using the ordination program CANOCO version 5.0 [57]. The RDA introduces a series of explanatory (environmental) variables and resembles the model of multivariate multiple regression, allowing us to determine what linear combinations of these explanatory variables determine the gradients. Data were centered, standardized, and log transformed fish trophic biomass by MPA. Explanatory variables consisted of MPA age, MPA size, distance from closest land, live coral cover, and benthic habitat characteristics [PC1, PC2]). PC1 and PC2 from the benthic PCA were used as variables to describe the benthic community among MPAs. To rank explanatory MPA variables in their importance for being associated with the structure of the fish assemblages, we used a forward selection where the statistical significance of each variable was judged by a Monte-Carlo unrestricted permutation test with 499 permutations [58].

## Results

### Benthic communities

Benthic community composition was not significantly different between MPAs and adjacent open areas (PERMANOVA pseudo-F_1,127_ = 0.44, p = 0.81, Table 2). Hard coral accounted for 50.6% (± 21.7 sd) of the overall benthic cover, followed by bare substrate (15.3% ± 15.1), CCA (9.1% ± 9.6), blue-green algae (6.2% ± 13.4), and macroalgae (6.0% ± 9.6). Based on SIMPER analysis, the average dissimilarity of benthic community composition between MPAs and open areas was only 33.4%. Although percent cover of hard coral was similar between MPAs and open areas (51.0 and 50.3%, respectively), it comprised 18.1% of the dissimilarity between management regimes. Bare substrate accounted for an additional 14.0% of the dissimilarity between management regimes, followed by blue-green algae (13.5), and CCA (11.9%).

**Table 2:** Comparison of benthic community composition among MPAs and adjacent open areas based on permutation-based multivariate analysis of variance (PERMANOVA).

Source	df	MS	Pseudo-F	P(perm)
Management	1	1092	0.44	0.813
Location(Management)	12	3155	8.37	0.001
Residuals	114	377		
Total	127			

**Fig 2:**
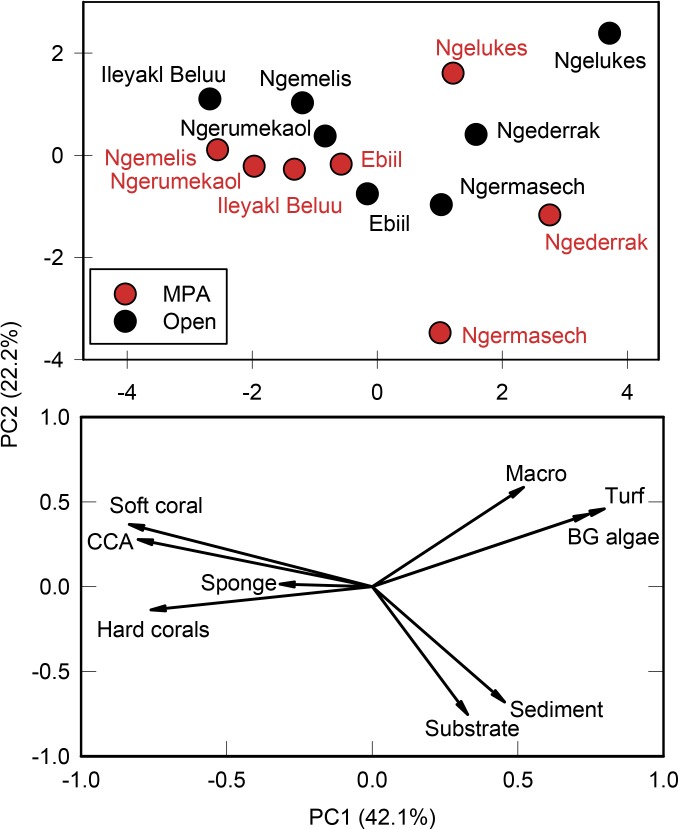
Principal component analysis (PCA) of major benthic groups from all sites. Percent cover data were arcsine square root transformed prior to analysis. Top figure shows site separation while lower figure shows drivers that explain the most variance in the principal components.

The first two principal component axes (PC1 and PC2) described over 64% of the variation in benthic cover data (Fig 2). Forereef MPAs (Ebiil, Ileyakl Beluu, Ngemelis, and Ngerumekaol) and their adjacent open sites clustered together in ordination space, while inshore areas (e.g., Ngelukes, Ngermasech, and Ngederrak) were distinct from the forereef areas and there was less concordance between paired protected and open sites within these inshore areas. PC1 described the gradient from offshore to inshore sites, with the major loadings being soft coral, CCA, and coral in the offshore direction and turf algae, blue-green algae, and macroalgae loading towards the inshore areas. PC2 was weakly associated with management, with the major loadings being bare substrate and sediment towards the bottom of the biplot (MPAs), and algae (macroalgae, turf, blue-green) towards the top (open areas). Macroalgae was, on average, 46% higher in open areas compared to MPAs, although overall macroalgae cover was extremely low (~6%). Bare substrate was 33% higher in MPAs compared with open areas, and sediment was 89% greater inside MPAs although again, the overall cover of sediment was low (1.3% inside MPAs and 2.5% outside).

Coral cover was not significantly different between MPAs and adjacent unprotected sites (χ2 _1, 128_ = 0.46, p = 0.50), except for the Ngederrak MPA, which had coral cover nearly two times lower than the adjacent open area (χ2 _1, 24_ = 9.54, p = 0.002). We found the highest coral cover in the Ngerumekaol MPA (68.2%), Ngerumekaol open area (62.9%), Ngemelis MPA (55.5%), and Ileyakl Beluu MPA (55.3%). The lowest coral cover was in the Ngederrak MPA (21.5%), which was affected more severely by the typhoon in 2013 than the adjacent open area [59].

### Fishes

#### Fish biomass

**Fig 3:**
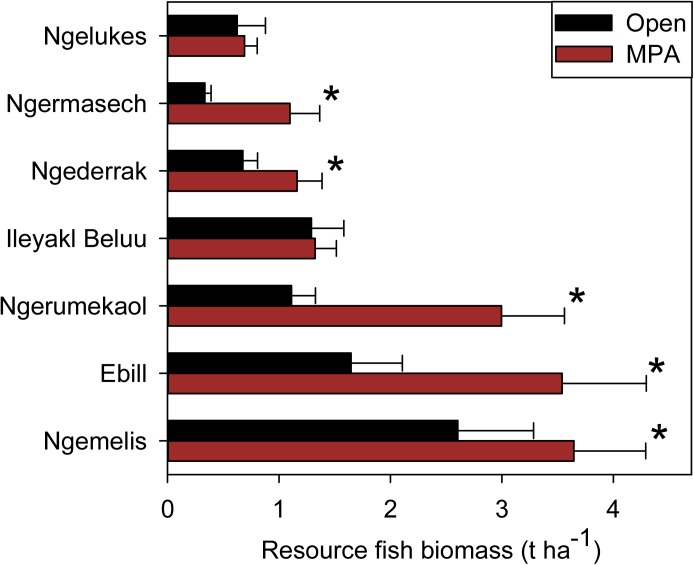
Comparison of resource fish biomass (t ha^-1^, mean ± standard error) inside and outside MPAs. Asterisks denote MPA/open pairs that are significantly different.

There were no significant differences in resource and non-resource fish biomass between depth strata (GLM, p > 0.05 for both), and samples were subsequently pooled. There was a highly significant difference in overall resource fish biomass between MPAs and open areas (χ2 _1, 384_ = 19.4, p < 0.001), but no significant difference in non-resource fish biomass (χ2 _1, 384_ = 0.20, p = 0.67). Resource fish biomass was significantly higher in five (Ebiil, Ngerumekaol, Ngederrak, Ngemelis, and Ngermasech) of the seven MPAs compared to their adjacent open areas (Fig 3). The most pronounced differences were found in the Ngermasech and Ngerumekaol MPAs, which had resource fish biomass 3.3 and 2.7 times higher, respectively, compared to their adjacent open areas. Variations in resource biomass within locations were relatively low, ranging from a CV of 11.6% at Ileyakl Beluu to 34.9% at Ngemelis

**Fig 4:**
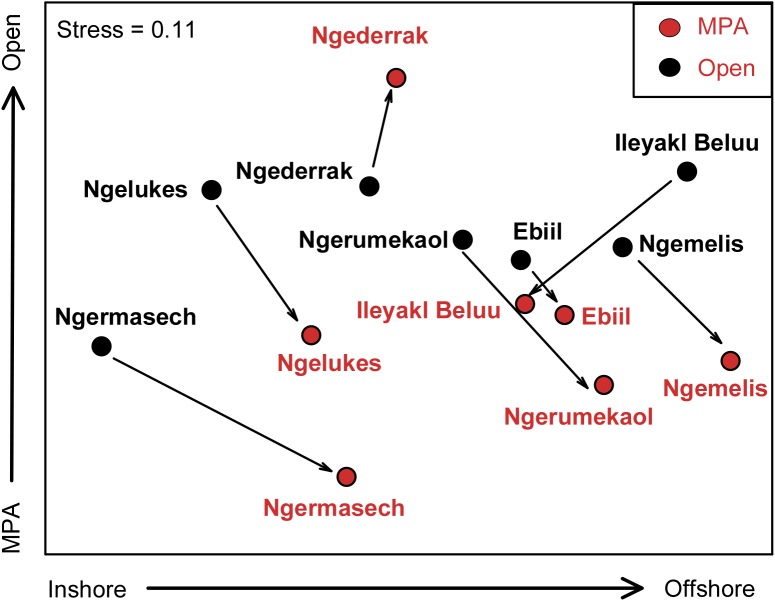
Nonmetric multidimensional scaling plot of mean fish biomass for each MPA and adjacent open areas. Arrows denote the direction and magnitude from open area to MPA in ordination space. Stress = 0.11.

Locations were well separated in ordination space based on fish species biomass (Fig 4). The first nMDS axis showed a strong gradient from nearshore to offshore locations moving from left to right along this axis. The second nMDS axis showed a gradient from MPAs to open areas moving from the bottom up along this axis, with the exception of the Ngederrak MPA, which was at the top of this axis.

#### Fish size and trophic structure

**Fig 5:**
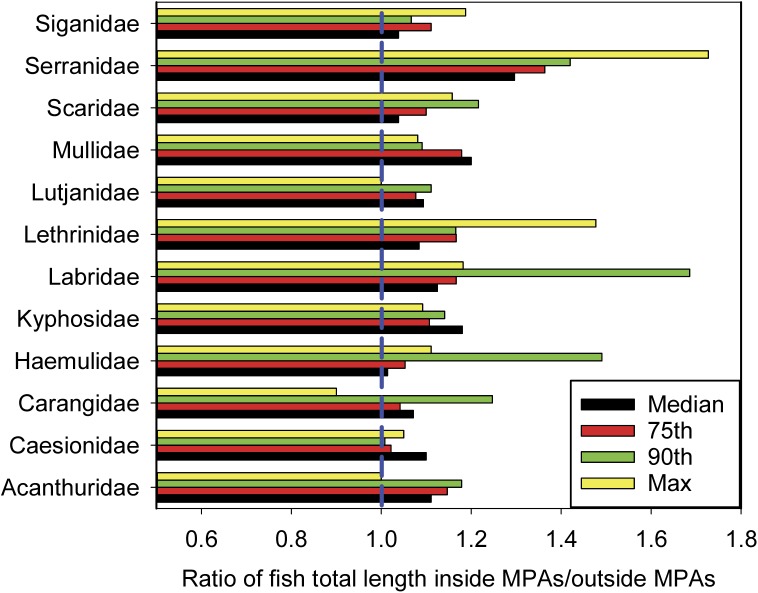
Ratio of fish lengths (TL) by family inside versus outside MPAs based on median, 75^th^ and 90^th^ percentiles, and maximum size.

Examination of fish sizes inside vs. outside MPAs showed larger lengths inside MPAs for median, 75^th^ and 90^th^ percentiles, and maximum size for nearly all major families of fishes surveyed (Fig 5). Wrasses (Labridae), groupers (Serranidae), emperors (Lethrinidae), and grunts (Haemulidae) showed the largest differences.

**Fig 6:**
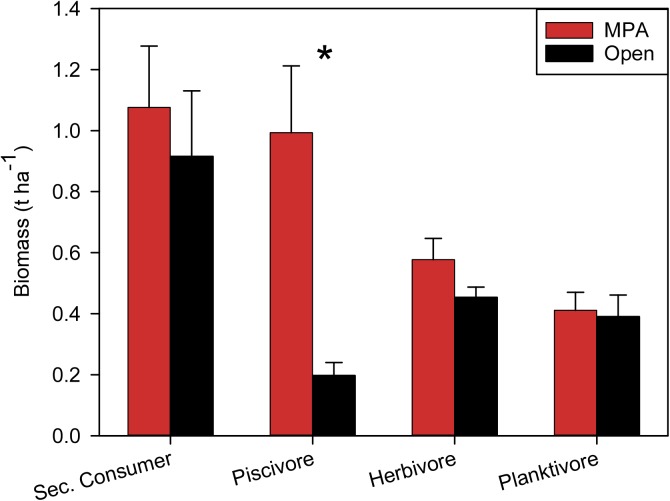
Biomass (t ha^-1^, mean ± standard error) by fish trophic groups and management (open to fishing and MPA). The asterisk identifies significant differences between MPA and adjacent open area.

The interaction between management and biomass by trophic group was significant (χ2 _1, 1536_ = 70.2, p < 0.001). Contrasts in biomass between MPAs and open areas within trophic groups showed highly significant differences for top predators (χ2 _1, 384_ = 78.9, p < 0.001), but not for any other trophic group (all p > 0.05) (Fig 6). Top predators accounted for 32.5% of the biomass in MPAs, but only 10% in adjacent open areas. Secondary consumers comprised 35% of the biomass inside MPAs and 47% in open areas. Herbivores accounted for 18.9% of the biomass inside MPAs and 23.2% in open areas. Planktivores comprised 13% of the biomass within MPAs and nearly 20% in open areas.

### Comparison of MPAs

**Fig 7:**
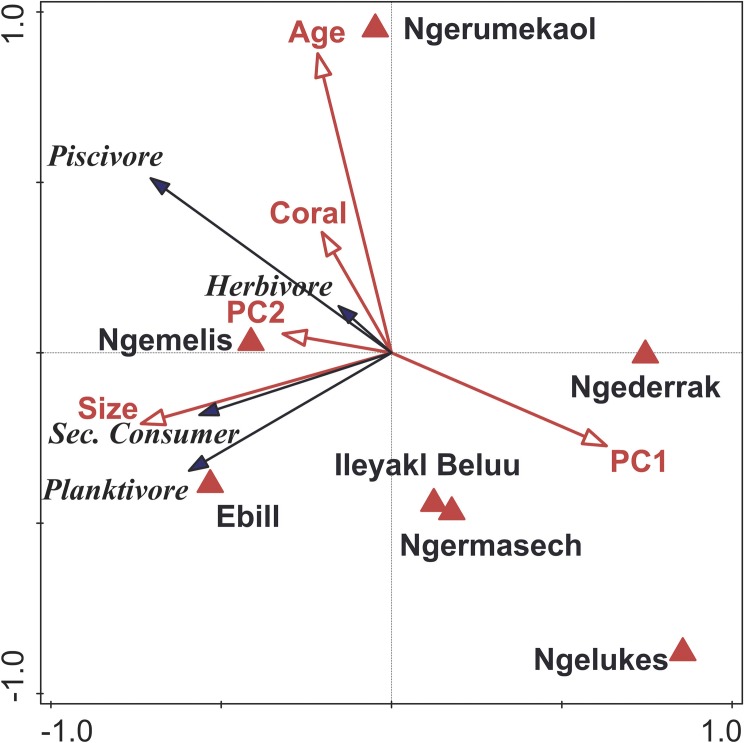
Biplot of results of redundancy analysis on fish biomass of trophic groups with MPA variables (MPA age, MPA size, distance from land, live coral cover, and benthic habitat [PC1, PC2]). Data were centered, standardized, and log transformed fish biomass for trophic groups by MPA. MPA characteristics were centered and standardized prior to analysis.

Our data show strong separation among MPAs based on fish trophic biomass (Fig 7, Table 3). The first two axes of the RDA biplot explained 53.5% of the trophic group variance and 96% of the trophic groups and MPA variables relationship (Table 3). In terms of trophic biomass structure, piscivores explained 50.0% of the cumulative fraction of variation explained by Axis 1, followed by planktivores, which explained an additional 26.2% of the cumulative variation. The only significant explanatory MPA variables involved in this ordination were MPA size and age, which were orthogonal to one another in ordination space. MPA size explained 52.2% of the variability in the fish trophic structure and MPA variable matrix, and separated MPAs along Axis 1. Years of protection (MPA age) explained 39.7% of the variability in this matrix and separated MPAs along Axis 2.

**Table 3:** A. Results of redundancy analysis (RDA) on log-transformed, fish biomass data for trophic groups with MPA variables (size, age, distance from land, coral cover, PC1, and PC2). B. Conditional effects of Monte-Carlo permutation results on the redundancy analysis (RDA).

A. Axes	Axis 1	Axis 2	Axis 3
Eigenvalues	0.38	0.15	0.01
Explained variation (cumulative)	38.38	53.53	54.78
Pseudo-canonical correlation	0.81	0.71	0.43
Explained fitted variation (cumulative)	80.83	92.22	97.11
B. Variable	Pseudo-F	P	% variance explained
MPA size	8.2	0.002	52.2
MPA age	7.6	0.002	39.7

## Discussion

The majority of the no-take MPAs in Palau surveyed during our expedition are effective in conserving resource fish biomass relative to adjacent fished sites. Resource fish biomass in Ngemelis and Ebiil (> 3 t ha^-1^) are comparable to that of pristine sites elsewhere in the Pacific [60–61]. The most striking difference in trophic structure between MPAs and fished areas was in the biomass of top predators (sharks, jacks, and groupers), which was 5 times larger in the MPAs compared to open areas. MPA size, and to a slightly lesser extent, age explained most of the variation in fish assemblage structure, particularly for piscivores, which are a major target of the local fisheries. Larger MPAs contain a greater amount and diversity of habitats, and have been shown to possess more and larger resource fishes compared with smaller MPAs [35, 45, 48]. The life history characteristics of coral reef fishes, especially for many large-bodied predators, are such that long-term (> 10 years) protection is necessary for fully recovery of populations [36, 46–48]. Several of the MPAs assessed in this study were specifically designed to protect these predator species, especially grouper spawning aggregations, which are particularly susceptible to overfishing [12, 62].

Palau possesses some of the best preserved and managed coral reefs remaining in the western Pacific [63–64], where much of the world’s marine biodiversity lies [65]. The level of enforcement of these MPAs is high, by most standards, due to strong local community support and patrolling [13]. Conservation rangers were present at every MPA we surveyed and there is general support for the PAN in Palau [13].

The use of traditional ecological knowledge in the establishment of Palau’s PAN has provided a customary framework to support western management, thereby creating greater acceptance by the local communities who manage these MPAs. The PAN consists of a wide variety of habitats and management regimes, ranging from complete no-take to subsistence fishing only. While our results only pertain to fully protected areas in the PAN, they may also have implications for other protected areas in the network.

There were no differences in coral cover and benthic community structure between MPAs and adjacent unprotected areas, therefore the greater abundance of resource fish inside MPAs is likely due to protection and not to differences in the state of the benthic communities. We did not detect differences in non-resource fish biomass, providing further evidence for the positive effects of protection from fishing. This highlights the fact that fishing, rather than other anthropogenic influences (e.g., pollution, habitat degradation) or intrinsic differences in local productivity or habitat quality, is likely primarily responsible for the observed differences in fish biomass between MPAs and adjacent areas open to fishing.

The habitat at forereef sites, for both MPAs and open areas, was dominated by CCA and hard coral, while turf algae, blue-green algae, and macroalgae characterized the inshore areas. Although macroalgae cover was low overall, it was nearly twice as high in open areas and may partially be in response to the higher herbivorous fish biomass in the MPAs compared with the open areas. Inshore areas, particularly around the large island of Babeldaob, suffer from the effects of sedimentation and pollution [4, 7, 66]. Both MPAs and areas open to fishing in these inshore areas had lower coral cover and high cover of macroalgae compared with more offshore reefs. Despite the poor habitat quality, inshore MPAs performed better than inshore areas open to fishing in terms of accumulating resource fish biomass. Reducing the effects of sedimentation and pollution in these inshore areas will likely improve fish biomass within these MPAs, as well as the areas open to fishing [10].

While our results are only a snapshot in time, they indicate that the no-take MPAs in Palau that we surveyed are meeting the goal of conservation of resource fishes. MPAs benefit adjacent fisheries by protecting large spawning individuals and through the spillover of adults into fished areas [67–70]. Networks of MPAs provide an option for increasing the ecological and economic benefits often provided by single MPAs [71]. The effectiveness of Palau’s extensive network of MPAs may likely benefit the nearshore fisheries of the entire country and improve the resilience of coral reefs by reducing their vulnerability to global climate change, and promote rapid recovery from natural impacts such as typhoons [58].

A comprehensive study by Houk et al. [10] used a robust and consistent methodology to examine the coral reef ecosystem condition in six jurisdictions across Micronesia: (i) the Marshall Islands, the states of (ii) Kosrae, (iii) Pohnpei, (iv) Chuuk, and (v) Yap, which comprise the Federated States of Micronesia, and (vi) Commonwealth of the Northern Mariana Islands. Using a number of biological metrics of fish and benthic assemblage structure, they found that only 42% of the major reef habitats examined exceeded the ecosystem-condition threshold of 70% established by the Micronesia Challenge [10]. MPAs in these jurisdictions showed little influence when grouped together across the region, emphasizing the limited amount of area currently located within MPAs in these other locations and the need for increased protection and better management, similar to those adopted by Palau.

Palau generates substantial income from tourism. A recent economic study in Palau showed that divers would be willing to pay more for diving in no-take MPAs because of more and larger fishes [72]. The economic benefits of more protection of just two charismatic species (Napoleon wrasse [*maml*] and bumphead parrotfish [*kemedukl*], currently protected in Palau) would be 100 to 1,000 times greater than the market value if those species were fished [72]. In addition, the value of live sharks in the water brings in $1.9 million to Palau’s economy through dive tourism, compared to $10,800 if these sharks were killed for sale [15]. These results suggest that greater levels of protection may bring greater economic revenue to Palau and could provide a model for other Pacific islands.

MPA effectiveness in Palau has been enhanced through the use of traditional knowledge combined with expert science and the development of MPA networks. Ownership, legacy, stewardship, and responsibility are essential elements of Palau’s approach to resource management and conservation [4]. Traditional approaches were, and still are, effective in managing human impacts on coral reefs and related resources in Palau [1, 73], and model legislation (Palau’s Marine Protection Act of 1994) was based on this traditional knowledge for protecting specific spawning sites and establishing fisheries closures.

While Palau’s MPAs are doing well relative to nearby areas open to fishing, previous work on spawning aggregation closures [12] and communications with fishermen indicate that fish abundance in Palau was much greater in the past. While in the ecosystem health of Palau’s MPAs are likely below historical baselines, they represent a step in the right direction towards recovery of the marine ecosystem, which is so critical to Palau and its people. The recent creation of the Palau National Marine Sanctuary protects ~500,000 km^2^ of its offshore waters, representing 80% of the country’s EEZ [74]. The protection provided by this new, large MPA around Palau could support increased diving tourism revenues, improve local fisheries, and ensure the long-term sustainability of marine resources.

## Supporting information

S1 Table List of fish species and their families used in surveys. Primary resource species denoted as yes. Main diet categories: herbivore, planktivore, secondary consumer, and piscivore.
(DOCX)
