# Supplementary material for: Size, age, and habitat determine effectiveness of Palau's Marine Protected Areas
Source: PLoS One. 2017 Mar 30;12(3):e0174787. doi: 10.1371/journal.pone.0174787 (PMC5373616; doi:10.1371/journal.pone.0174787)
Supplement: S1 Table — Primary resource species denoted as yes. Main diet categories: herbivore, planktivore, secondary consumer, and piscivore. (DOCX) [file pone.0174787.s001.docx]

S1 Table. List of fish species and their families used in surveys. Primary resource species denoted as yes. Main diet categories: herbivore, planktivore, secondary consumer, and piscivore.

| Family | Scientific name | Primary resource species | Main diet |
| --- | --- | --- | --- |
| Acanthuridae | *Acanthurus blochii* | Yes | Herbivore |
| Acanthuridae | *Acanthurus leucocheilus* | Yes | Herbivore |
| Acanthuridae | *Acanthurus lineatus* | Yes | Herbivore |
| Acanthuridae | *Acanthurus nigricans* | Yes | Herbivore |
| Acanthuridae | *Acanthurus nigrofuscus* | Yes | Herbivore |
| Acanthuridae | *Acanthurus nigroris* | Yes | Herbivore |
| Acanthuridae | *Acanthurus olivaceus* | Yes | Herbivore |
| Acanthuridae | *Acanthurus pyroferus* | Yes | Herbivore |
| Acanthuridae | *Acanthurus* sp. | No | Herbivore |
| Acanthuridae | *Acanthurus thompsoni* | No | Planktivore |
| Acanthuridae | *Acanthurus triostegus* | Yes | Herbivore |
| Acanthuridae | *Acanthurus xanthopterus* | Yes | Herbivore |
| Acanthuridae | *Ctenochaetus binotatus* | Yes | Herbivore |
| Acanthuridae | *Ctenochaetus cyanocheilus* | Yes | Herbivore |
| Acanthuridae | *Ctenochaetus striatus* | Yes | Herbivore |
| Acanthuridae | *Naso annulatus* | Yes | Planktivore |
| Acanthuridae | *Naso brachycentron* | Yes | Planktivore |
| Acanthuridae | *Naso brevirostris* | Yes | Herbivore |
| Acanthuridae | *Naso caesius* | Yes | Planktivore |
| Acanthuridae | *Naso hexacanthus* | Yes | Planktivore |
| Acanthuridae | *Naso lituratus* | Yes | Herbivore |
| Acanthuridae | *Naso tonganus* | Yes | Herbivore |
| Acanthuridae | *Naso unicornis* | Yes | Herbivore |
| Acanthuridae | *Naso vlamingii* | Yes | Planktivore |
| Acanthuridae | *Paracanthurus hepatus* | No | Planktivore |
| Acanthuridae | *Zebrasoma scopas* | No | Herbivore |
| Acanthuridae | *Zebrasoma veliferum* | No | Herbivore |
| Caesionidae | *Caesio caerulaurea* | No | Planktivore |
| Caesionidae | *Caesio cuning* | No | Planktivore |
| Caesionidae | *Caesio lunaris* | No | Planktivore |
| Caesionidae | *Caesio teres* | No | Planktivore |
| Caesionidae | *Pterocaesio lativittata* | No | Planktivore |
| Caesionidae | *Pterocaesio marri* | No | Planktivore |
| Caesionidae | *Pterocaesio pisang* | No | Planktivore |
| Caesionidae | *Pterocaesio tile* | No | Planktivore |
| Caesionidae | *Pterocaesio trilineata* | No | Planktivore |
| Carangidae | *Alectis ciliaris* | Yes | Piscivore |
| Carangidae | *Carangoides bajad* | Yes | Piscivore |
| Carangidae | *Carangoides ferdau* | Yes | Piscivore |

S1 Table. List of fish species and their families used in surveys. Continued

| Family | Scientific name | Primary resource species | Main diet |
| --- | --- | --- | --- |
| Carangidae | *Carangoides fulvoguttatus* | Yes | Piscivore |
| Carangidae | *Carangoides orthogrammus* | Yes | Piscivore |
| Carangidae | *Carangoides plagiotaenia* | Yes | Piscivore |
| Carangidae | *Caranx ignobilis* | Yes | Piscivore |
| Carangidae | *Caranx melampygus* | Yes | Piscivore |
| Carangidae | *Caranx sexfasciatus* | Yes | Piscivore |
| Carangidae | *Elagatis bipinnulata* | Yes | Piscivore |
| Carangidae | *Scomberoides lysan* | Yes | Piscivore |
| Carcharhinidae | *Carcharhinus amblyrhynchos* | Yes | Piscivore |
| Carcharhinidae | *Carcharhinus melanopterus* | Yes | Piscivore |
| Carcharhinidae | *Negaprion acutidens* | Yes | Piscivore |
| Carcharhinidae | *Triaenodon obesus* | Yes | Secondary consumer |
| Chanidae | *Chanos chanos* | Yes | Herbivore |
| Echeneidae | *Echeneis naucrates* | No | Secondary consumer |
| Haemulidae | *Plectorhinchus albovittatus* | Yes | Secondary consumer |
| Haemulidae | *Plectorhinchus chaetodonoides* | Yes | Secondary consumer |
| Haemulidae | *Plectorhinchus chrysotaenia* | Yes | Secondary consumer |
| Haemulidae | *Plectorhinchus gibbosus* | Yes | Secondary consumer |
| Haemulidae | *Plectorhinchus lessonii* | Yes | Secondary consumer |
| Haemulidae | *Plectorhinchus lineatus* | Yes | Secondary consumer |
| Haemulidae | *Plectorhinchus orientalis* | Yes | Secondary consumer |
| Haemulidae | *Plectorhinchus vittatus* | Yes | Secondary consumer |
| Kyphosidae | *Kyphosus cinerascens* | Yes | Herbivore |
| Kyphosidae | *Kyphosus* sp. | Yes | Herbivore |
| Labridae | *Bodianus axillaris* | No | Secondary consumer |
| Labridae | *Bodianus mesothorax* | No | Secondary consumer |
| Labridae | *Cheilinus fasciatus* | No | Secondary consumer |
| Labridae | *Cheilinus trilobatus* | No | Secondary consumer |
| Labridae | *Cheilinus undulatus* | Yes | Secondary consumer |
| Labridae | *Choerodon anchorago* | Yes | Secondary consumer |
| Labridae | *Epibulus insidiator* | No | Secondary consumer |
| Lethrinidae | *Gnathodentex aureolineatus* | No | Secondary consumer |
| Lethrinidae | *Lethrinus atkinsoni* | Yes | Secondary consumer |
| Lethrinidae | *Lethrinus erythracanthus* | Yes | Secondary consumer |
| Lethrinidae | *Lethrinus erythropterus* | Yes | Secondary consumer |
| Lethrinidae | *Lethrinus harak* | Yes | Secondary consumer |
| Lethrinidae | *Lethrinus lentjan* | Yes | Secondary consumer |
| Lethrinidae | *Lethrinus obsoletus* | Yes | Secondary consumer |
| Lethrinidae | *Lethrinus olivaceus* | Yes | Secondary consumer |

S1 Table. List of fish species and their families used in surveys. Continued

| Family | Scientific name | Primary resource species | Main diet |
| --- | --- | --- | --- |
| Lethrinidae | *Lethrinus* sp. | Yes | Secondary consumer |
| Lethrinidae | *Lethrinus xanthochilus* | Yes | Secondary consumer |
| Lethrinidae | *Monotaxis grandoculis* | Yes | Secondary consumer |
| Lethrinidae | *Monotaxis heterodon* | Yes | Secondary consumer |
| Lutjanidae | *Aphareus furca* | Yes | Piscivore |
| Lutjanidae | *Aprion virescens* | Yes | Piscivore |
| Lutjanidae | *Lutjanus argentimaculatus* | Yes | Secondary consumer |
| Lutjanidae | *Lutjanus biguttatus* | Yes | Planktivore |
| Lutjanidae | *Lutjanus bohar* | Yes | Piscivore |
| Lutjanidae | *Lutjanus fulvus* | Yes | Secondary consumer |
| Lutjanidae | *Lutjanus gibbus* | Yes | Secondary consumer |
| Lutjanidae | *Lutjanus kasmira* | Yes | Piscivore |
| Lutjanidae | *Lutjanus monostigma* | Yes | Piscivore |
| Lutjanidae | *Lutjanus semicinctus* | Yes | Secondary consumer |
| Lutjanidae | *Lutjanus* sp. | Yes | Secondary consumer |
| Lutjanidae | *Macolor macularis* | No | Secondary consumer |
| Lutjanidae | *Macolor niger* | No | Secondary consumer |
| Lutjanidae | *Symphorichthys spilurus* | Yes | Secondary consumer |
| Mullidae | *Mulloidichthys vanicolensis* | Yes | Secondary consumer |
| Mullidae | *Parupeneus barberinoides* | Yes | Secondary consumer |
| Mullidae | *Parupeneus barberinus* | Yes | Secondary consumer |
| Mullidae | *Parupeneus crassilabris* | Yes | Secondary consumer |
| Mullidae | *Parupeneus cyclostomus* | Yes | Piscivore |
| Mullidae | *Parupeneus indicus* | Yes | Secondary consumer |
| Mullidae | *Parupeneus multifasciatus* | Yes | Secondary consumer |
| Mullidae | *Parupeneus pleurostigma* | Yes | Secondary consumer |
| Muraenidae | *Gymnothorax javanicus* | No | Piscivore |
| Myliobatididae | *Aetobatus narinari* | No | Secondary consumer |
| Scaridae | *Bolbometopon muricatum* | Yes | Secondary consumer |
| Scaridae | *Cetoscarus bicolor* | Yes | Herbivore |
| Scaridae | *Chlorurus bleekeri* | Yes | Herbivore |
| Scaridae | *Chlorurus japanensis* | Yes | Herbivore |
| Scaridae | *Chlorurus microrhinos* | Yes | Herbivore |
| Scaridae | *Chlorurus sordidus* | Yes | Herbivore |
| Scaridae | *Hipposcarus longiceps* | Yes | Herbivore |
| Scaridae | *Scarus altipinnis* | Yes | Herbivore |
| Scaridae | *Scarus chameleon* | Yes | Herbivore |
| Scaridae | *Scarus dimidiatus* | Yes | Herbivore |
| Scaridae | *Scarus flavipectoralis* | Yes | Herbivore |
| Scaridae | *Scarus forsteni* | Yes | Herbivore |

S1 Table. List of fish species and their families used in surveys. Continued

| Family | Scientific name | Primary resource species | Main diet |
| --- | --- | --- | --- |
| Scaridae | *Scarus frenatus* | Yes | Herbivore |
| Scaridae | *Scarus ghobban* | Yes | Herbivore |
| Scaridae | *Scarus globiceps* | Yes | Herbivore |
| Scaridae | *Scarus niger* | Yes | Herbivore |
| Scaridae | *Scarus oviceps* | Yes | Herbivore |
| Scaridae | *Scarus prasiognathos* | Yes | Herbivore |
| Scaridae | *Scarus psittacus* | Yes | Herbivore |
| Scaridae | *Scarus rubroviolaceus* | Yes | Herbivore |
| Scaridae | *Scarus schlegeli* | Yes | Herbivore |
| Scaridae | *Scarus* sp. | Yes | Herbivore |
| Scaridae | *Scarus spinus* | Yes | Herbivore |
| Scombridae | *Gymnosarda unicolor* | Yes | Piscivore |
| Scombridae | *Sarda orientalis* | Yes | Piscivore |
| Scombridae | *Scomberomorus commerson* | Yes | Piscivore |
| Serranidae | *Aethaloperca rogaa* | Yes | Piscivore |
| Serranidae | *Anyperodon leucogrammicus* | Yes | Piscivore |
| Serranidae | *Cephalopholis argus* | Yes | Piscivore |
| Serranidae | *Cephalopholis boenak* | Yes | Piscivore |
| Serranidae | *Cephalopholis cyanostigma* | Yes | Piscivore |
| Serranidae | *Cephalopholis leopardus* | Yes | Piscivore |
| Serranidae | *Cephalopholis miniata* | Yes | Piscivore |
| Serranidae | *Cephalopholis sexmaculata* | Yes | Piscivore |
| Serranidae | *Cephalopholis spiloparaea* | Yes | Piscivore |
| Serranidae | *Cephalopholis urodeta* | Yes | Piscivore |
| Serranidae | *Cromileptes altivelis* | Yes | Piscivore |
| Serranidae | *Epinephelus coeruleopunctatus* | Yes | Secondary consumer |
| Serranidae | *Epinephelus fuscoguttatus* | Yes | Piscivore |
| Serranidae | *Epinephelus malabaricus* | Yes | Secondary consumer |
| Serranidae | *Epinephelus melanostigma* | Yes | Piscivore |
| Serranidae | *Epinephelus merra* | Yes | Secondary consumer |
| Serranidae | *Epinephelus polyphekadion* | Yes | Secondary consumer |
| Serranidae | *Gracila albomarginata* | Yes | Piscivore |
| Serranidae | *Plectropomus areolatus* | Yes | Piscivore |
| Serranidae | *Plectropomus laevis* | Yes | Piscivore |
| Serranidae | *Plectropomus leopardus* | Yes | Piscivore |
| Serranidae | *Variola louti* | Yes | Piscivore |
| Siganidae | *Siganus argenteus* | Yes | Herbivore |
| Siganidae | *Siganus corallinus* | Yes | Herbivore |
| Siganidae | *Siganus doliatus* | Yes | Herbivore |
| Siganidae | *Siganus puellus* | Yes | Herbivore |

S1 Table. List of fish species and their families used in surveys. Continued

| Family | Scientific name | Primary resource species | Main diet |
| --- | --- | --- | --- |
| Siganidae | *Siganus punctatissimus* | Yes | Herbivore |
| Siganidae | *Siganus punctatus* | Yes | Herbivore |
| Siganidae | *Siganus vulpinus* | Yes | Herbivore |
| Sphyraenidae | *Sphyraena barracuda* | Yes | Piscivore |
| Sphyraenidae | *Sphyraena forsteri* | Yes | Piscivore |
| Sphyraenidae | *Sphyraena qenie* | Yes | Piscivore |
| Zanclidae | *Zanclus cornutus* | No | Secondary consumer |
